# Robotic Bronchoscopy: Review of Three Systems

**DOI:** 10.3390/life13020354

**Published:** 2023-01-28

**Authors:** Maxwell J. Diddams, Hans J. Lee

**Affiliations:** 1 Division of Pulmonary and Critical Care, University of North Carolina at Chapel Hill, Chapel Hill, NC 27514, USA; 2 Division of Pulmonary and Critical Care, School of Medicine, Johns Hopkins University, Baltimore, MD 21287, USA; hlee171@jhmi.edu

**Keywords:** bronchoscopy, interventional pulmonology, robotic bronchoscopy, robotics, lung cancer, navigational bronchoscopy, pulmonary nodule

## Abstract

Robotic bronchoscopy (RB) has been shown to improve access to smaller and more peripheral lung lesions, while simultaneously staging the mediastinum. Pre-clinical studies demonstrated extremely high diagnostic yields, but real-world RB yields have yet to fully matched up in prospective studies. Despite this, RB technology has rapidly evolved and has great potential for lung-cancer diagnosis and even treatment. In this article, we review the historical and present challenges with RB in order to compare three RB systems.

## 1. Introduction

Robotic bronchoscopy (RB) for the diagnosis and treatment of peripheral pulmonary lesions has become a topic of intense focus since the first robotic bronchoscopy platform was FDA-approved in 2018. Early clinical pilot studies suggested that RB would be a highly accurate platform for pulmonary nodule management [1,2,3], thanks to machine-controlled stability and integrated navigation for the precise evaluation of even sub-centimeter nodules. Further research has called into question the extremes of these claims [4,5], but it has not dampened the enthusiasm for robotic-assisted intervention into the bronchoscopy suite. In this article, we review the development of RB, the current three systems in the US, the advantages and disadvantages, and the future directions for RB, through a PubMed search of all articles with the terms “robotic” and “bronchoscopy”.

## 2. The Dilemma of Pulmonary Nodules and Early-Stage Lung Cancer

Lung cancer is the number one cause of cancer deaths worldwide, causing 25% of all cancer-related deaths [6]. Despite increased screening, 66% of lung cancers are still diagnosed at a late stage [7]. Small pulmonary nodules (SPN) are increasing in incidence on computed tomography (CT) [8]. These nodules are increasingly detected on incidental scans, as well as via lung cancer screening, and the risk these nodules present for patients has been challenging to quantify [8,9]. Many risk-stratification algorithms have been designed, based on nodule and patient characteristics [10]. These prediction models derive from specific patient populations and, when applied appropriately, they can reliably predict the likelihood of nodule malignancy. If a nodule is high-risk, then intervention without a biopsy is appropriate; if it is sufficiently low-risk, then observation is advised [11]. Many nodules will fall into an intermediate category, where biopsy is often necessary, but even in high-risk patients, most of these nodules will be benign. For example, in the National Lung Cancer Screening Trial (NLST), 96% of positive scans were false positives [12]. Furthermore, results from the Checkmate 816 trial have shown significant increases in event-free survival for resectable stage-Ib-IIIa lung cancers treated with checkpoint inhibitors and chemotherapy prior to surgery, so accurate pre-surgical sampling is critical for improved patient outcomes.

Non-surgical lung biopsies for SPNs have largely taken two forms—transthoracic needle aspiration (TTNA) and bronchoscopic. TTNA biopsy with fluoroscopic guidance was first described in 1965 [13]; it has undergone significant developments with CT, ultrasound, and electromagnetic guidance. Recent retrospective reviews of CT-guided TTNA biopsies indicate a diagnostic accuracy of approximately 67–97%, even for ground-glass nodules less than 2 cm in size [14]. Only moderate or local sedation is usually needed, sparing the patient the risk of general anesthesia. The most common complications are pneumothorax and hemorrhage, which occur in approximately 20% and 3% of cases, respectively. One-third of patients with pneumothorax have been reported to need chest-tube placement [14]. While TTNA provides a highly accurate sampling method, its pneumothorax rate, its inability to stage the mediastinum, and its limitation to the outer region of the lung are its major drawbacks.

The use of flexible fiberoptic bronchoscopy for the biopsy of SPNs has been largely limited to central, larger lesions. Lesions less than 2 cm in size, in particular, have had diagnostic yields as low as 14% when located in the peripheral third of the lung and 31% when located in the inner two-thirds [15]. Flexible bronchoscopy is usually performed under moderate sedation with airway topicalization, and major complications are rare. The most common complications, such as mild, transient hypotension or hypoxemia, are usually associated with moderate sedation. Respiratory depression, bleeding, arrythmia, pneumothorax, and cardiorespiratory arrest occur in less than 1% of cases, and transbronchial biopsy increases these risks, including bleeding and pneumothorax, to approximately 2–4% [16]. Mortality is exceptionally rare—as low as 0.013% in one retrospective study of 23,862 patients [17].

While bronchoscopy is clearly safe, its diagnostic limitations led to the development of navigational bronchoscopy. For years, navigation could be carried out with painstaking review of CT images and in-suite fluoroscopy, but in 1996, radial endobronchial ultrasound (rEBUS) became commercially available for the ultrasonographic evaluation of peripheral nodules [18]. The rEBUS probe could be inserted through the working channel of the bronchoscope and target lesions could be visualized by their relatively hypoechoic and heterogenous appearance, compared to that of the normal aerated lung. While rEBUS was useful in confirming the localization of the target lesion prior to biopsy, navigating to the correct airway was still a significant challenge.

An initial foray into direct navigational guidance was virtual bronchoscopy [19]. CT images were synthesized into on-screen images of the airways to assist the bronchoscopist in navigating the correct airways to the target lesion. Initially, these images were static images on an adjacent screen, which the bronchoscopist could manually advance along a pre-specified pathway in parallel to their visualized airways [20]. In 2004, the SuperDimension electromagnetic navigation bronchoscopy (ENB) platform was released. It used an electromagnetic field generator to track the movement of the bronchoscope throughout the airways. Once a same-day CT scan was registered, the ENB system not only provided active guidance through the airways, but also a virtual simulation of the nodule’s location relative to the tip of the bronchoscope [21]. This active tool-tracking and live-updating navigation, akin to the GPS in a car, largely marked the advent of true navigational bronchoscopy.

Compared to flexible bronchoscopy, navigational bronchoscopy was able to achieve a much higher diagnostic yield. Initial prospective studies of the SuperDimension system reported an 80% diagnostic yield in 75% peripheral SPNs, 57% of which were less than 2 cm. The pneumothorax rate was 3.5% [21]. In the 2018 multi-center prospective NAVIGATE trial, the diagnostic yield for malignancy using an ENB system, with or without rEBUS or fluoroscopy, was 72.9%, although the definition of diagnostic yield has come into question, as indeterminate pathology results were included in the true negative group at 12 months of follow-up. That said, more than half of these SPNs were less than 2 cm in size, in the upper lobes, and in the outer third of the lung. Despite a quarter of these lesions lying on the visceral pleura, the pneumothorax rate was 4.3% overall, with only 2.9% requiring hospitalization or intervention [22]. Similar success was reported for other ENB systems, as well as for fluoroscopic and cone-beam guided navigational bronchoscopy systems [23,24].

Unfortunately, follow-up studies on EMN were not as forgiving. While the positive predictive value in the NAVIGATE trial was 100%, the negative predictive value was 47–64%, similar to that of rEBUS alone [22]. Registries of diagnostic yield in real-world practice, outside of single-center exemplars and large, high-volume centers, began to show significant heterogeneity. For example, the AQuIRE registry showed a diagnostic yield of traditional bronchoscopy, rEBUS, and EMN of 63.7%, 57%, and 38.5%, respectively, suggesting that the success of EMN in the community was likely overreported by publication bias [25]. Diagnostic yield across these studies was not uniformly defined, leading to variable reporting. In addition to these technical challenges, navigational bronchoscopy required the bronchoscopist to maintain millimeter-level stability for prolonged periods. Given the high risk of patient harm from a false-negative biopsy, the stage was set for RB platforms.

## 3. Robotic Bronchoscopy

The path to RB parallels the development of robotic surgery. In thoracic surgery, open thoracotomy has been associated with significant morbidity, especially for older, frailer patients [26,27]. Studies on thoracotomy in the elderly in the 1980s showed an in-hospital mortality of 4–17%. Complications, including air leaks, arrhythmia, and secretions, were seen in 37% of patients, and 5-year survival following lung cancer resection was 27% overall [28]. Other studies of elective thoracotomy in octogenarians correlated mortality with comorbidities, suggesting that outcomes are likely limited by heavy selection bias [29]. Video-assisted thoracoscopic surgery (VATS) significantly reduced lengths of stay, arrhythmias, days with chest tubes, transfusions, kidney injuries, pneumonia, and prolonged air leaks, compared to open thoracotomy for lobectomy [30,31]. Robotic-assisted thoracoscopic surgery (RATS) is associated with similar outcomes to those of VATS, but it has improved confined space movement, tremor suppression, 3D optics, and ergonomics [32]. RATS is associated with significantly higher costs than VATS, especially in the initial investment [32]. While these benefits are now apparent, the proof and acceptance of RATS has required decades of careful research.

With the release of the Monarch™ platform (Auris Health, Inc., Redwood City, CA, USA) in 2018, bronchoscopy began along a similar path. Each of the RB platforms to date use a proprietary omnidirectional bronchoscope mounted on a robotic arm and navigate via a pre-procedure CT scan that is registered to the bronchoscope’s location at the beginning of the procedure. The cost is still significant for all the systems, including initial purchase, maintenance, and processing fees. Similar to robotic surgery, RB was no longer dependent on the physician’s hands, and pre-clinical cadaveric studies with the Monarch system demonstrated 100% navigational success [1], access to twice as many bronchial divisions than that of standard flexible bronchoscopy of equal outer diameter (8 vs. 4) [33], and a 97% diagnostic yield [34]. Shortly thereafter in 2019, the Ion™ endoluminal RB platform (Intuitive Surgical©, Sunnyvale, CA, USA) received FDA clearance, distinguishing itself with a shape-sensing bronchoscope. In contrast to the Monarch system, which used an electromagnetic field to maintain orientation in space, the Ion system uses fiberoptic bend sensors within the catheter itself to maintain orientation. Four years later, the Galaxy System™ (Noah Medical, San Carlos, CA, USA), which is expected to have FDA clearance in 2023, provides EMN and digital tomosynthesis guidance on top of a disposable bronchoscope. A summary of the comparative technical specifications are provided in Table 1.

### 3.1. Monarch™ Platform (Auris Health, Inc., Redwood City, CA, USA)

**Figure 1:**
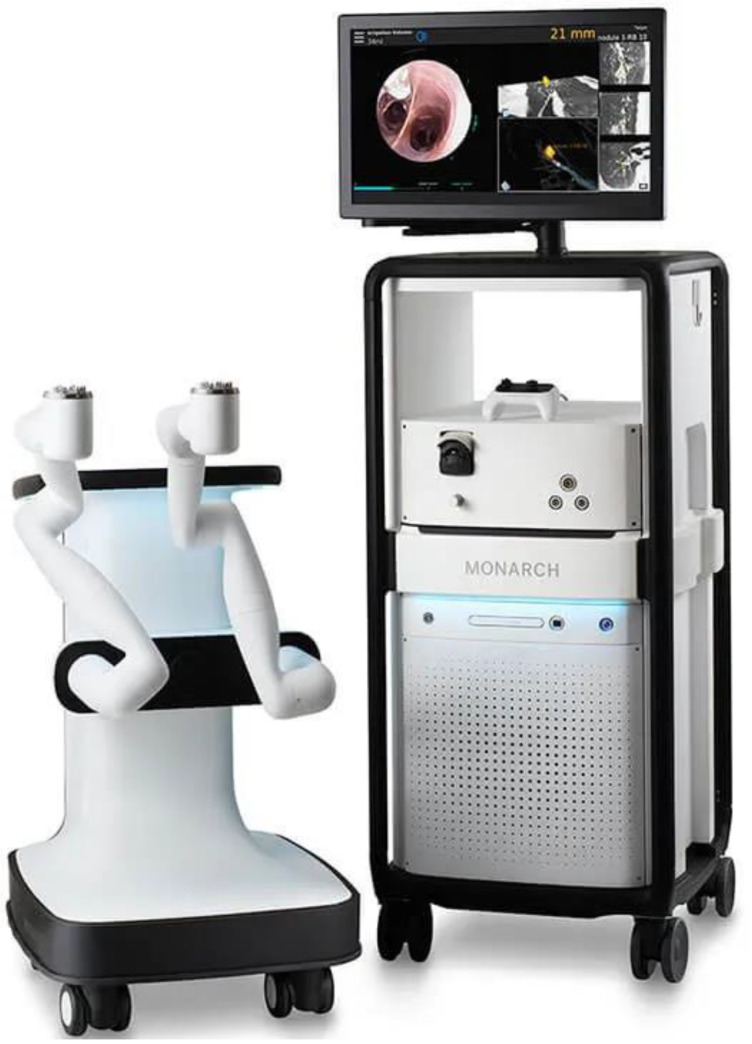
Monarch™ platform (Images courtesy of Auris Health, Inc., Redwood City, CA, USA).

The Monarch™ RB platform (Figure 1) uses an articulating bronchoscope within an articulating sheath, each of which is controlled by independent robotic arms mounted on the RB cart. A separate tower with a monitor screen and a controller connect to the RB cart. The outer sheath has a 6.0 mm outer diameter (OD), while the scope itself has a 4.4 mm OD, a 2.1 mm diameter working channel (WC), and an integrated camera. The outer sheath can articulate up to 130 degrees in any direction, and the inner sheath can be individually advanced and articulated up to 180 degrees in any direction. The system is fundamentally an electromagnetic navigation system referenced against sensors applied to the patient’s chest, and the bronchoscopy cart has a small enough footprint to allow for a C-arm or Cone-beam CT (CBCT). The system monitor can integrate vision, navigation, rEBUS, and CT overlay, and it is controlled with a video-game-style controller with two thumb-sticks.

### 3.2. Ion™ Endoluminal RB Platform (Intuitive Surgical©, Sunnyvale, CA, USA)

**Figure 2:**
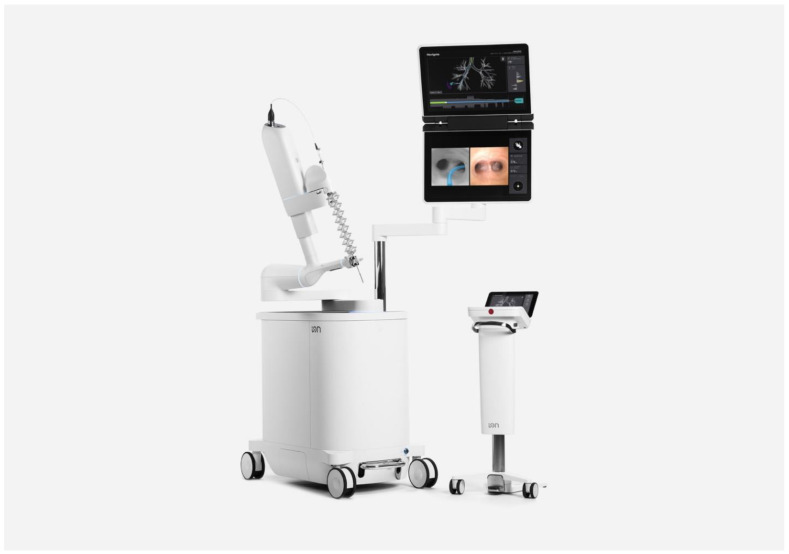
Ion Robotic Bronchoscopy System (Images courtesy of Intuitive Surgical©, Sunnyvale, CA, USA).

The Ion™ RB system (Figure 2) uses a single ultrathin bronchoscope with integrated, proprietary shape-sensing technology to localize and maintain scope position within the airway. The bronchoscope as a 3.5 mm OD with a 2 mm WC, but the vision probe must be removed from the working channel to insert other tools. The catheter can articulate up to 180 degrees in any direction and is controlled by a ball mouse and scroll wheel. The robotic arm controlling the scope and the system monitor are both mounted on the same bronchoscopy cart, and the monitor can simultaneously show navigation, fluoroscopy, virtual overlay, and either vision or rEBUS. The system footprint is small enough to work with fluoroscopy and CBCT, and the lack of EMN obviates interference concerns.

### 3.3. Galaxy System™ (Noah Medical, San Carlos, CA, USA)

**Figure 3:**
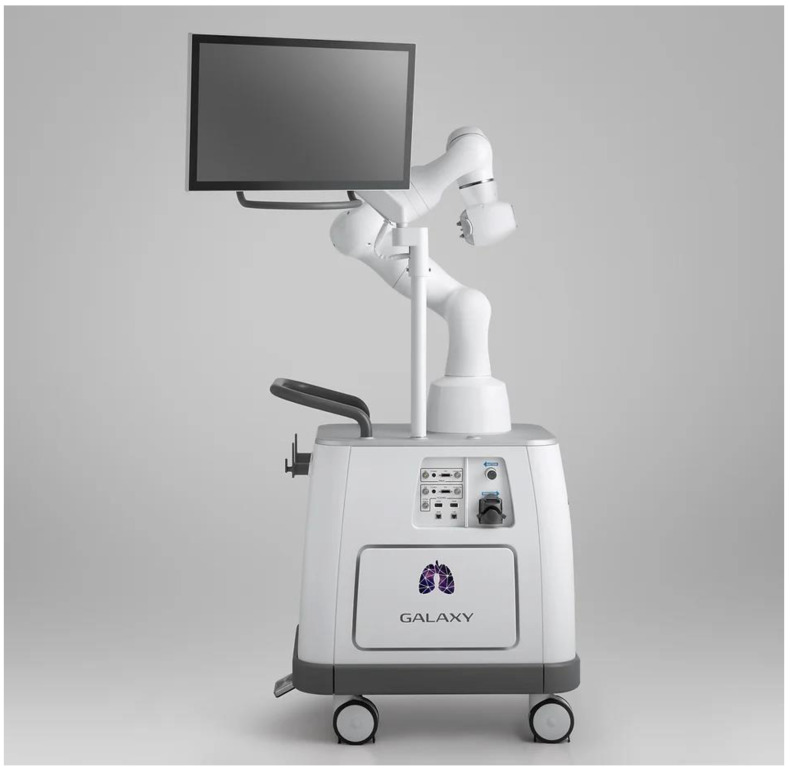
Galaxy System™ (Images courtesy of Noah Medical, San Carlos, CA, USA).

The Galaxy System™ (Figure 3) does not have FDA approval at the time of writing, but it is being evaluated. Billing itself as a more cost-competitive RB platform, the system promises an EMN-guided single-use bronchoscope with 4.0 mm OD, 2.1 mm WC, and integrated vision. To overcome the localization limitations of EMN, the system uses EMN to navigate to within 2 cm of the target lesion, then uses proprietary digital tomosynthesis (TiLT+ Technology™) via any standard fluoroscopy C-arm to confirm tool-in-lesion. Digital tomosynthesis is an imaging technique similar to CT scanning, where a series of X-ray images are taken from different angles to reconstruct a 3D image. Unlike CT scans, digital tomosynthesis takes fewer images from a relatively narrower angle (15–60 degrees, compared to 180 degrees for CT) [35], but as a result it can be performed with a standard C-arm rather than a dedicated CT scanner. If there is divergence between the simulated target and the fluoroscopically observed target lesion, the operator can readjust, and in the case of fluoroscopically invisible lesions an augmented fluoroscopy mode can be used to overlay a simulated lesion on the fluoroscopy screen. The cart combines a monitor and a non-proprietary robotic arm for the smallest cart size of the systems, and it is controlled by a video-game-style controller. Cost is a major focus of the system, with off-the-shelf robotic arm components, no reprocessing for the disposable scope, and native fluoroscopy/CBCT integration advertised to provide consumer savings. The first human trial with the system is currently underway in Australia.

## 4. Advantages of Robotic Bronchoscopy

The main benefit of an RB system has been replacing human hands, which are subject to fatigue, with finely controlled computerized systems to achieve more accurate navigation and tool delivery. The first single center feasibility study of the Monarch™ system showed a 93% localization rate in 15 patients [36], and in a follow-up 5-center observational trial of 55 patients, this localization rate was borne out at 96% [37]. Initial pilot studies of the Ion™ system reported similar localization rates of 85–96.6% [38,39], and both studies reported low rates of pneumothorax, at 0–5.8%, with roughly half of those patients requiring chest tube placement [36,38,39,40]. Bleeding rates in these studies were low, at 2.4–3.2% [4,5,41]. Fluoroscopy and rEBUS were used in most of these cases, and CBCT was used in a smaller subset.

An underappreciated feature of RB systems has been their navigation software. Navigation pathway generation software with the Ion™ system has been shown to be superior to EMN in both the distance from the terminal end of the navigation pathway to target lesions (9.4 mm for robotic bronchoscopy vs. 14.2 mm for tip-tracked electromagnetic navigation vs. 17.2 mm for catheter-based electromagnetic navigation) and the generation of complete distal airway maps [42]. With RB, proximity to the target lesion has been shown to be the strongest predictor of a central hit on the lesion, independent of the presence of a bronchus sign, divergence, or concentric rEBUS view [43].

With this excellent localization and complication rate, a similarly improved diagnostic yield would be expected, but results have been mixed. Feasibility studies for both systems have shown a diagnostic yield of 69–79% [4,39], and only small, single-center reports were able to post diagnostic yields in the 96% range [3]. Noting the comparable diagnostic yield of 72.9% reported from the prospective-multicenter pre-RB NAVIGATE trial, many investigators appropriately asked if anything had changed with the addition of these costly systems [44]. The response, in the EMN era, has been the integration of additional imaging modalities to provide tool-in-lesion confirmation. CBCT and various advanced fluoroscopy systems have been trialed, and these have shown increased diagnostic yields in the 86–94% range, though without direct comparison to other non-RB modalities [38,40,45]. Wile the numerical trends are encouraging, there is significant variation in the definitions of diagnostic yield, and only one head-to-head trial has compared the effect of these modalities. Low et al. (2022) published the only comparison to date of EMN with digital tomosynthesis versus shape-sensing bronchoscopy alone, which showed equivalent diagnostic yields of 80% and 77%, respectively [46].

Of note, RB has been compared to TTNA for diagnostic yield. A retrospective multicenter review of 225 patients in the Mayo Clinic Health System, equally split between TTNA and RB, showed overall diagnostic yields of 87.6% for RB and 88.4% for TTNA. Complication rates were significantly higher for TTNA than for RB (17% vs. 4.4%) [47]. The average cost for an admission for primary spontaneous pneumothorax has been retrospectively estimated at $13,961 ± $15,789, with total costs of $52,118 ± $69,490 [48], and this does not include the cost of the staging bronchoscopy that is required for positive lesions. Future prospective studies will likely be able to demonstrate cost benefit over, or at least cost parity with, TTNA at the system level.

## 5. Limitations

A key technical issue that has not been definitively solved is that of CT-to-body divergence. RB has definitively improved the bronchoscopists ability to navigate to the target lesion, as defined by the navigation software, but when tested by rEBUS, fluoroscopy, or CBCT, there is a margin of error between the simulated target and the end of the bronchoscope [49]. This difference between the static pre-procedural CT image and the changing, mobile lung during the procedure is termed CT-to-body divergence [50]. For example, Benn et al. demonstrated a diagnostic yield of 86% with RB and CBCT, but had to reposition their bronchoscope under CBCT guidance 15% of the time to ensure that the biopsy tool was in the lesion [38]. When rEBUS shows no lesion at the supposed target, diagnostic yield drops from 84–85% to 38% [5]. While opportunities for improved navigational planning likely exist, the discrepancy between the virtual targets generated and the actual lesion location likely relate to anatomical changes in the lung from atelectasis and movement. The time from intubation to significant atelectasis in the prospective I-LOCATE trial was 30 min, which was well within the timeframe of an RB procedure with a preceding endobronchial ultrasound transbronchial needle aspiration [51]. Mucous plugging or pleural effusions can cause transient atelectasis, and differences in patient positioning between CT and OR can contribute to divergence as well [50].

Several potential solutions have been proposed to improve CT discordance, including increasing positive end-expiratory pressure during bronchoscopy to 10–12, increasing tidal volumes to 10–12 mL per kg of ideal body weight, expeditious intubation, use of paralytics, using a low fraction of inspired oxygen to reduce absorptive atelectasis, avoidance of excess suction, breath holds for CBCT spins, and minimizing procedure times to less than 30 min [52,53]. Controversially, some authors have advocated starting with navigational bronchoscopy first, rather than mediastinal staging as recommended by current guidelines, as a possible solution [54]. Prospective studies are needed to evaluate these solutions, as well as to establish best practices.

Minimum procedure requirements and formal skill certification may be necessary. For example, consider VATS lobectomy. Compared to open thoracotomy, VATS lobectomy has a significantly different surgical approach and technique. The learning curve for each individual surgeon varies, and many thoracic surgeons were initially concerned that VATS resulted in a sub-optimal cancer staging procedure [55]. Ultimately, VATS has been proven to have fewer complications and shorter lengths of stay, particularly in elderly patients [55,56]. Many surgeons report needing more than 50 VATS lobectomies to become comfortable with the procedure, and some authors have recommended that a VATS lobectomy program should include performing more than 25 lobectomies a year [55]. In one center, the rate of conversion from VATS to open thoracotomy decreased from 28% to 11% over a 3-year period, as VATS cases significantly increased [57]. Similarly, surgeons who had already performed at least 20 robotic mitral valve repairs required an average of 250 operations before their complication rates dropped from 25% to 10%, and 75–125 operations just to overcome their learning curve [58]. RB combines many technologies that are familiar to bronchoscopists, but they still have a significant learning curve to achieve competency. RB appears to be less mentally demanding than ENB in cognitive-load studies of bronchoscopists experienced with EMN [59], but there is likely a wide variation in time-to-competency. In EMN, this has been shown for fellows and experienced bronchoscopists [60,61], and this variability in skill-acquisition needs to be described for RB moving forward.

RB companies provide significant teaching support, but it is unclear when a proceduralist should be considered competent or how best to assess skill acquisition. From a consumer safety perspective, there is no standardized summative assessment to ensure competency, and as diagnostic yield comes further into focus it is important to note that competency needs to include mastery of the imaging tools necessary to maintain excellent diagnostic accuracy, not just competency in driving the bronchoscope. Although it may be a barrier to RB platform sales, a standardized summative assessment of RB competency would be in patients’ best interests.

Unfortunately, robotic systems are expensive, and no open publications on initial cost, maintenance, and processing are currently available for comparison. Proprietary tools are often required, and no analysis has been published on factors such as tool use and type per case, OR time, or even general cost efficacy. Such information will be critical to the appropriate uptake or concentration of RB, as well for as its generalizability to low- and middle-income settings.

## 6. Discussion

The rapid development of RB has outpaced the ability of high-quality studies to describe procedural characteristics and outcomes. Decreased diagnostic yields from CT-to-body divergence exemplify this issue, as publications on its remedy with CBCT were published before actual full-sized prospective cohort studies on RB itself [37]. The concept of visualized tool-in-lesion as equivalent to diagnostic yield has proven to be fraught, with one prospective study by Benn et al., with 100% of cases demonstrating tool-in-lesion, only producing an 86% diagnostic yield [38]. Since CBCT and advanced fluoroscopy systems make up a significant cost that is additional to the already expensive RB systems, it is critical to accurately demonstrate the utility of these systems. In the interim, improvements in biopsy tool design and pathology evaluation techniques may resolve the remaining gap in diagnosis.

Fundamental to the question of how to improve diagnostic yield and accuracy is the question of how to define these terms. Diagnostic yield is the likelihood that a test will establish a diagnosis (number of biopsies with a specific diagnosis divided by total biopsies), while diagnostic accuracy is the number of biopsies with the correct final diagnosis. While histopathology demonstrating malignancy or characteristic benign pathology proves a clear hit on the target lesion, labeling findings, such as non-specific inflammation, non-diagnostic atypia, or normal lung, as diagnostic for benign disease can artificially inflate diagnostic yield. Differences in definitions may contribute to apparent marginal gains of RB diagnostic yield, and uniform definitions are necessary for accurate comparisons moving forward [46,62,63].

Given that 60% of NSCLC are adenocarcinomas requiring next-generation sequencing, measuring the diagnostic yield of RB for molecular diagnosis rather than just for malignant/non-malignant disease will also be critical. The adequacy for NGS with radial EBUS trans-bronchial biopsy for peripheral lesions was 97.9% in one prospective study [64], and EBUS TBNA was similarly high at 77–95%, depending on the number of passes [65,66]. Peripheral biopsy of metastatic lesions was reported to have a molecular diagnostic yield of 98% for those patients tested in one prospective observational study of EMN for peripheral nodules [67]. Similarly, adequacy for molecular analysis was 100% in another prospective observational study, but the diagnostic yield was 48–63% [68]. Studies comparing the molecular yield of RB systems will be critical and dependent on definitions of diagnostic yield.

With these limitations in mind, comparisons of RB systems are limited, but generate optimism. Shape-sensing RB appeared to have a similar diagnostic yield to that of digital tomosynthesis-assisted EMN in the one prospective comparative study that was carried out [46]; however, whether the addition of CBCT or other advanced imaging to shape-sensing bronchoscopy would change this comparison is unknown. Direct pricing comparisons are not possible based on public data that are currently available, but competitively priced RB platforms are likely to be of interest in the absence of clear comparative performance data. Integration with existing OR space and radiology equipment is likely to be a key driver of choice.

## 7. Future Directions

Overcoming divergence is a critical next step in RB. Augmented imaging such as cone-beam and advanced fluoroscopy may be key to resolving this problem, and multiple studies are underway. Endobronchial microscopy techniques, such as confocal laser microscopy, may improve localization [69,70]. New biopsy techniques are likely needed, given previous reports of sub-perfect diagnostic yield, despite 100% tool-in-lesion confirmation. Research in steerable needles [71], RB mounted cryobiopsy probes for peripheral biopsy [72], and the application of next-generation sequencing to small tissue biopsies [73] may ultimately help close the diagnostic gap.

Cost and reimbursement developments are desperately needed. Despite lung cancer being the number one cause of cancer deaths, most community hospitals may struggle to justify the high costs of both a new bronchoscopy platform and its corequisite advanced imaging system. This is due in part to direct purchase and maintenance costs, but also largely due in part to low current reimbursement for bronchoscopic procedures. For example, in 2022, Medicare did not have a dedicated CPT code for RB, but only the 31,627 add-on code for EMN [74]. Outside of wealthier countries, these upfront costs will likely remain a large barrier to the widespread adoption of RB for years to come. Within the US, advocacy for bronchoscopic payment reform is needed to help fund lung cancer centers of excellence.

With the tool stability of RB, there is intense interest in therapeutic bronchoscopy [75]. Multiple human trials for the endobronchial ablation of lung cancers are currently enrolling on clinicaltrials.gov, with a variety of microwave, radiofrequency, thermal, laser, and CryoSpray ablation techniques. Efforts have also focused on direct intratumoral injection of chemotherapy and other agents to directly enhance immunotherapy in the local tumor microenvironment [76]. RB may have an important role in peri-operative nodule marking for minimally invasive thoracic surgery [77]. More revolutionarily, with the results of JCOG 0802 showing the benefit of segmentectomy over lobectomy for stage IA non-small-cell lung cancer [78], RB may be poised to finally facilitate a single-procedure diagnosis, stage, and cure for early-stage lung cancer. While the advantage of RB therapeutics will not be certain for many years, what is certain is that RB developments will continue to outstrip the research describing them.

## 8. Conclusions

RB is still following the path of robotic surgery in the early phase of proof-of-benefit, without robust studies. The performance characteristics and complication rates of the two RB systems on the market, appear to be similar, and RB seems to be able to achieve high levels of diagnostic yield in specialized, high-volume centers with a high prevalence of malignancy. Moving forward, large, multicenter, prospective trials are needed to accurately quantify the diagnostic yield and procedural characteristics of RB, RB systems, and their adjunct tools. At this time, the racing pace of innovation makes that quantification challenging.

## Figures and Tables

**Table 1:** Technical Differences in Robotic Bronchoscopy Platforms.

	Monarch Robotic Bronchoscopy System (Auris Health, Inc., Redwood City, CA, USA)	Ion Robotic Bronchoscopy System (Intuitive Surgical©, Sunnyvale, CA, USA)	The Galaxy System (Noah Medical, San Carlos, CA, USA)
Navigation Technology	Electromagnetic Navigation	Shape Sensing	Electromagnetic with digital tomosynthesis TiLT+ Technology™
Catheter Outer Diameter	Outer Sheath: 6 mm Inner Scope: 4.2 mm	3.5 mm	4.0 mm
Working Channel Diameter	2.1 mm	2 mm	2.1 mm
Vision during Biopsy	Yes	No	Yes
Scope Reprocessing	Yes	Yes	No, disposable
Compatibility with Cone Beam or Advanced Fluoroscopy	Yes	Yes	Yes
Therapeutic tools	Under Investigation	Under Investigation	Unclear
FDA Approval	Yes	Yes	Pending

## Data Availability

Data sharing not applicable.
